# Association of Elevated Plasma FGF21 and Activated FGF21 Signaling in Visceral White Adipose Tissue and Improved Insulin Sensitivity in Gestational Diabetes Mellitus Subtype: A Case-Control Study

**DOI:** 10.3389/fendo.2021.795520

**Published:** 2021-11-29

**Authors:** Ning Wang, Bo Sun, Haonan Guo, Yingyu Jing, Qi Ruan, Mengjun Wang, Yang Mi, Huan Chen, Lin Song, Wei Cui

**Affiliations:** ^1^ Department of Endocrinology and Second Department of Geriatrics, The First Affiliated Hospital of Xi’an Jiaotong University, Xi’an, China; ^2^ Department of Physiology and Pathophysiology, School of Basic Medical Sciences, Xi’an Jiaotong University Health Science Center, Xi’an, China; ^3^ Department of Endocrinology, 521 Hospital of Norinco Group, Xi’an, China; ^4^ The Second Department of Obstetrics, Northwest Women and Children’s Hospital, Xi’an, China

**Keywords:** gestational diabetes mellitus, heterogeneity, fibroblast growth factor 21, insulin sensitivity, adipose

## Abstract

**Objective:**

To study the discrepancy of the insulin sensitivity alteration pattern, circulating fibroblast growth factor (FGF21) levels and FGF21 signaling in visceral white adipose tissue (vWAT) of gestational diabetes mellitus (GDM) subtypes.

**Methods:**

26 GDM women with either a predominant of insulin-secretion defect (GDM-dysfunction, n = 9) or insulin-sensitivity defect (GDM-resistance, n = 17) and 13 normal glucose tolerance (NGT) women scheduled for caesarean-section at term were studied. Blood and vWAT samples were collected at delivery.

**Results:**

The insulin sensitivity was improved from the 2^nd^ trimester to delivery in the GDM-resistance group. Elevated circulating FGF21 concentration at delivery, increased FGF receptor 1c and decreased klotho beta gene expression, enhanced ERK1/2 phosphorylation, and increased GLUT1, IR-B, PPAR-γ gene expression in vWAT were found in the GDM-resistance group as compared with the NGT group. The circulating FGF21 concentration was negatively correlated with fasting blood glucose (r = -0.574, *P* < 0.001), and associated with the GDM-resistance group (r = 0.574, *P* < 0.001) in pregnant women at delivery. However, we observed no insulin sensitivity alteration in GDM-dysfunction and NGT groups during pregnancy. No differences of plasma FGF21 level and FGF21 signaling in vWAT at delivery were found between women in the GDM-dysfunction and the NGT group.

**Conclusions:**

Women with GDM heterogeneity exhibited different insulin sensitivity alteration patterns. The improvement of insulin sensitivity may relate to the elevated circulating FGF21 concentration and activated FGF21 signaling in vWAT at delivery in the GDM-resistance group.

## Introduction

Gestational diabetes mellitus (GDM) is a common obstetric diseases during pregnancy with spontaneous hyperglycemia during the second and third trimesters without pre-gestational diabetes mellitus (1). GDM increases the risk for a variety of maternal metabolic diseases and adverse perinatal outcomes for the infant, such as postpartum type 2 diabetes mellitus (2), macrosomia and shoulder dystocia (3). Women with GDM can be divided into subtypes based on insulin secretion and sensitivity heterogeneity (4). In clinical studies, we found differences in the risk factors of GDM subtypes (5), and having large-for-gestational-age infants is associated with specific GDM subtypes (6).

Fibroblast growth factor 21(FGF21) is a pleiotropic hormone-like protein regulates glucose and lipid metabolism (7), such as increasing tissue glucose uptake, improving insulin sensitivity, and inhibiting lipolysis (8). FGF21 is mainly produced by liver, and acts on target organs such as liver, adipose tissue and skeletal muscle (7, 9). The main peripheral site of FGF21 that promotes glucose uptake is the white adipose tissue (WAT) (10).

Many findings focused on the relationship between FGF21 and GDM lack consistency (9, 11–13). Circulating FGF21 concentration was positively associated with many clinical insulin resistance markers in pregnant women (14), and plasma FGF21 level at early pregnancy is considered as a potential predictor of GDM (15, 16). Wang and colleagues found the increased plasma FGF21 level at early 2^nd^ trimester were associated with higher probability of the diagnosis of GDM at the 24^th^ to 28^th^ gestational week, and significant statistically differences in FGF21 levels were existed based on different stratification of BMI (16). However, BMI could not fully reflect the pathophysiological process leading to hyperglycemia of GDM women. The relationships of the circulation FGF21 levels and the GDM heterogeneity on pathophysiological aspect, and insulin sensitivity alterations during pregnancy in women with GDM remain obscure.

We aimed to elucidate the relevance of insulin sensitivity alteration pattern in GDM subtypes to the circulating FGF21 concentration at delivery, to further investigate the FGF21 signaling in visceral WAT (vWAT) at delivery, and hope to provide individualized FGF21-targeted treatment for GDM women according to their specialized subtype.

## Methods

### Participants

Pregnant Chinese women were recruited from the Northwest Women and Children’s Hospital (NWCH) for participation in this case-control study. The participants were scheduled for delivery by caesarean-section from DEC. 2019 to MAR. 2020. Clinical information was collected from the 2^nd^ trimester study visit (24^th^ to 28^th^ weeks of gestation) until delivery. The 75-g, 2 h oral glucose tolerance test (OGTT) were performed at the 24^th^ – 28^th^ gestational weeks, and GDM was diagnosed based on the criteria of the International Association of Diabetes and Pregnancy Study Groups (17). The exclusion criteria was that: diabetes before pregnancy; fasting blood glucose (FBG) ≥ 7.0 mmol/L, 2-h glucose ≥ 11.1 mmol/L during OGTT; HbA1c ≥ 6.5% in the first trimester; eclampsia; young maternal age (less than 18); multiple pregnancy; other pre-existing diseases or gestational complications; and loss of vital data. Finally, from a total of 120 pregnant women, 57 subjects met the criteria and were included in further analysis.

### Clinical Measurements and Definitions

Pre-pregnancy body mass index (pre-BMI), maternal age at delivery, and the gestational weight gain (GWG), gestational age at delivery and birth weight were noted. ISI composite index and the Stumvoll I index were adopted to evaluate the insulin sensitivity and insulin secretion, respectively. The information used for the calculation is obtained from the OGTT and the simultaneous insulin-release test (18–20). Women with normal glucose tolerance were defined as the control group (NGT, n = 13). We classified GDM subtypes by Powe’s definition (4), which is based on the distributions of the ISI composite index and the Stumvoll I index in the NGT group. GDM women with insulin sensitivity defect were defined if the ISI composite index was under the 25^th^ percentile of the range in the NGT group. GDM women with insulin secretion defect were defined if the Stumvoll I index was under the 25^th^ percentile of the range in the NGT group. Due to the limited number of participants in this study, we used the indexes of the women in the NGT group of our previous study (5) to calculate the normal range, since participants in both studies came from the same population over the same time periods. Therefore, we divided GDM women into the two subgroups: GDM with a primary defect of insulin-sensitivity (GDM-resistance, n = 17), GDM with a primary defect of insulin-secretion (GDM-dysfunction, n = 9). We excluded patients who had all the stated characters, or had the two indexes over the 25^th^ percentile.

### The Collection of Blood Sample and Adipose Specimen

The fasting blood samples were collected using the EDTA-coated tubes (Sarstedt, Newton, NC). At delivery, maternal blood was collected and centrifuged at 1000g for 15 minutes at 4°C. Then, the plasma was stored at -80°C for following assays. The vWAT specimen was obtained from the greater omentum (visceral) during caesarean-section, quickly frozen in liquid nitrogen and reserved at -80°C for following studies.

### The Biochemical Parameters of Plasma

FBG was detected by the glucose oxidase approach (intra-assay variation factor was 2.1% and inter-assay variation factor was 2.6%). Plasma lipid profiles were detected using enzyme catalyzed approach according to the manufacturer’s procedure (A110-1-1, A111-1-1, A113-1-1, A112-1-1, Nanjing Jiancheng, China). Levels were quantified by a Microplate Reader with the wavelength of 546nm. The lipid levels included plasma triacylglycerol (TG), total cholesterol (CHO), low-density lipoprotein cholesterol (LDL-C) and high-density lipoprotein cholesterol (HDL-C). Plasma insulin levels were detected using commercial available kits (R-C-01-01, 5–180μU/mL, 3V Bioengineering, China). All lab tests were conducted in the certified lab of the NWCH with standard laboratory methods.

### Quantitative Plasma FGF21 Measurement

The plasma FGF21 levels at delivery were tested *via* a purchasable ELISA kit (CSB-E16844 h, Cusabio Biotech, Wuhan, China). All measurements were conducted according to the manufacturers’ protocol. The reference range of this detection was 15.6–1000 pg/mL with a susceptibility of 3.9 pg/mL. The coefficients of variability intra-assay and inter-assay were < 8% and < 10%, respectively.

### The Calculation of Insulin Sensitivity and Insulin Secretion Indexes


$$\text{ISI composite index}=\frac{10000}{\surd (\text{FBG}\times \text{FINS})\times (\text{average GLU}\times \text{average INS})}$$

(21)

GLU and INS measurements depicted as mmol/L and uU/mL, separately.


$$\begin{array}{c} \text{Stumvoll I index}=2032+4.681\times \text{INS}0-135.0\times \text{GLU}120+0.995\\ \times \text{INS}120+27.99\times \text{BMI}-269.1\times \text{GLU}0 \end{array}$$

(20)

GLU and INS survey depicted as mmol/L and uU/mL, separately. We used the HOMA2-S and HOMA2-β at http://www.dtu.ox.ac.uk (11 Jan. 2016) as indexes to measure statuses of insulin sensitivity and insulin secretion.

### Quantitative Polymerase Chain Reaction (qPCR)

The TRIzol reagent (Invitrogen, CA, USA) was employed to isolate total RNA from vWAT. 1μg total mRNA was reverse-transcribed into cDNA with the RT-PCR Kit (Thermo Scientific, USA). The entire qPCR was conducted with the iQ5 PCR thermocycler (Bio-Rad, USA). The primer sequences for the tested genes were presented in **Supplementary Table 1**. The LightCycler protocol below was performed: 95°C for thirty seconds (pre-cultivation); 40 periods of 95°C for five seconds and 60°C for thirty seconds (amplify); and 81 periods of 55°C for ten seconds (melting curve). We included negative controls in the entire qPCR operations. The -△△Ct method was used to identify the comparative expressing scores. Each sample was analyzed in duplicate. Cyclophilin was used as the housekeeping gene. The efficiency of each primer was coherent within experiment groups.

### Western Blotting

The vWAT was homogenized with RIPA buffer (Beyotime, China) with the protease inhibitor and phosphatase inhibitor (Roche, Germany). Equal amount of protein was loaded in the 10% TGX stain-free gels (Bio-Rad) and was then transferred to PVDF membranes (Millipore). Images captured of stain-free gels were used to determine the total protein amount. The membranes were blocked with 5% non-fat dry milk and then were incubated with the primary antibodies at 4°C overnight. The Cell Signaling Technology offered antibodies below: phosphorylated and total protein kinase B [Akt (4723/4550)], phosphorylated and total extracellular signal-regulated kinase 1/2 [Erk1/2 (4695/4370)]. Then the membranes were treated with secondary antibodies at room temperature for 1h and processed for enhanced chemiluminescence detection. The ChemiDoc Touch Imaging System (Bio-Rad) was used to visualize the total protein quantity and the targeted protein signals. Image Lab software (Bio-Rad) was used to perform the densitometric assay of the total protein and the targeted protein signals in all lanes. The ratio of phosphorylated protein value to total protein value was employed to express the changes in protein activation.

### Statistical Analysis

Statistical analyses were done using SPSS 22.0 (SPSS Inc., USA). Data were displayed as means (SD or SEM) or median (IQR). One-way ANOVA was used for normal distribution data, the Kruskal–Wallis test was used for non-normal distribution continuous variables, and the Chi-squared test (or Fisher’s exact possibility test) was used for class variables to compare the differences across the three groups (the NGT group and the two GDM subgroups). Tukey’s test, Dunn’s test and Chi-squared test were carried out to conduct the pairwise comparisons between the NGT group and the experimental groups when the *P*-value from either of the above tests was < 0.05. The Bonferroni correction was used to modify the *P*-value for Chi-squared test paired contrasts. Regression model such as linear and multiple linear regression were applied to analysis the relationships of two continues variables.

## Results

### Clinical Characteristics of Women at the Second and Third Trimester

**Table 1** shows that women in the GDM-resistance group had higher pre-BMI (*P* = 0.006, **Table 1**) as compared with the NGT group. We observed no statistically significant differences in maternal age, GWG and infant birth weight among the three groups during pregnancy.

**Table 1:** Clinical characteristics of women in the second and third trimester.

	GDM-resistance	*P^a^*	GDM-dysfunction	*P^a^*	NGT
Number (n)	17		9		13
Maternal age (years)	32.17 ± 3.00	–	34.78 ± 2.99	–	31.07 ± 5.02
Family history of diabetes mellitus (n,%)	2 (11.8)	–	1 (11.1)	–	1 (7.7)
Pre-BMI (kg/m^2^)	25.87 ± 2.00	0.006	20.93 ± 1.69	–	22.47± 4.01
Gestational week (weeks)	38 ± 0.62	–	37 ± 0.91	–	38 ± 0.46
Smoking status (n,%)	0	–	0	–	0
Alcohol consumption (n,%)	0	–	0	–	0
GWG (kg)	11.85 ± 4.74	–	12.77 ± 3.41	–	15.32 ± 4.01
Infant birth weight (g)	3380.59 ± 505.87	–	3274.44 ± 391.32	–	3346.15 ± 447.89
**The second trimester**					
OGTT					
FBG (mmol/L)	5.28 ± 0.40	0.005	5.69 ± 0.90	< 0.001	4.61 ± 0.34
1h glucose OGTT (mmol/L)	8.93 ± 1.81	–	9.56 ± 1.93	0.04	7.77 ± 1.03
2h glucose OGTT (mmol/L)	7.82 ± 1.29	0.014	6.97 ± 1.31	–	6.57 ± 0.90
AUC (glucose)	15.48 ± 2.22	0.027	15.90 ± 2.85	0.024	13.36 ± 1.19
Fasting insulin (uU/mL)	17.35 ± 4.85	< 0.001	6.28 ± 1.61	–	7.81 ± 4.93
1-h insulin OGTT (uU/mL)	159.01 ± 40.43	< 0.001	34.33 ± 14.17	–	54.09 ± 24.94
2-h insulin OGTT (uU/mL)	142.00 (95.25-165.90)	< 0.001	26.00 (17.40-27.55)	0.040	35.80 (27.70-55.10)
AUC (insulin)	254.50 (204.31-278.92)	< 0.001	49.20 (30.83-64.55)	0.042	74.95 (59.44-86.06)
HOMA2-β	162.17 ± 32.33	0.003	76.37 ± 24.56	0.018	118.27 ± 39.74
HOMA2-S	40.8 (32.35-48.60)	0.001	90.40 (82.35-133.80)	–	101.34 (81.65-111.00)
Insulin sensitivity (ISI composite index)	38.48 (32.35-48.60)	< 0.001	135.92 (101.00-174.89)	–	135.05 (100.72-168.92)
Insulin secretion (Stumvoll I index)	1164.25 (732.58-1416.11)	< 0.001	77.73 (18.30-98.14)	0.002	675.85 (456.09-774.16)
**Before delivery**					
OGTT					
FBG (mmol/L)	4.50 ± 0.12	–	5.33 ± 0.44	<0.001	4.38 ± 0.15
1h glucose OGTT (mmol/L)	8.65 ± 1.21	–	8.98 ± 1.23	0.029	7.84 ± 1.00
2h glucose OGTT (mmol/L)	7.13 ± 0.96	–	6.91 ± 1.36	–	6.46 ± 0.87
AUC (glucose)	14.47 ± 1.50	0.035	15.10 ± 1.82	0.020	13.26 ± 1.23
Fasting insulin (uU/mL)	15.29 ± 3.58	< 0.001	5.75 ± 1.77	–	7.74 ± 4.58
1-h insulin OGTT (uU/mL)	143.51 ± 38.81	< 0.001	32.84 ± 13.84	0.021	53.30 ± 24.73
2-h insulin OGTT (uU/mL)	132.20 (82.05-155.30)	< 0.001	25.00 (17.10-26.00)	0.027	34.30( 26.85-53.00)
AUC (insulin)	222.2 (176.6-255.38)	< 0.001	48.21 (35.76-68.98)	0.029	71.80 (59.05-83.13)
HOMA2-β	196.44 ± 39.45	< 0.001	75.37 ± 19.60	0.006	128.92 ± 42.96
HOMA2-S	46.70 (38.60-59.20)	0.001	103.60 (90.30-169.25)	–	109.90 (82.85-151.65)
Insulin sensitivity (ISI composite index)	47.00 (40.40-62.30)	< 0.001	136.98 (101.03-193.48)	–	139.11 (106.68-176.56)
Insulin secretion (Stumvoll I index)	1183.10 (731.70-1410.60)	< 0.001	74.16 (16.76-92.11)	< 0.001	660.79 (402.17-748.70)
TG (mmol/L)	3.79 ± 1.24	–	3.92 ± 2.13	–	3.66 ± 0.79
CHO (mmol/L)	5.61 ± 1.17	–	6.71 ± 1.10	–	6.09 ± 1.90
HDL-C (mmol/L)	1.69 ± 0.87	–	1.79 ± 0.38	–	2.08 ± 0.68
LDL-C (mmol/L)	2.86 ± 0.82	0.033	3.93 ± 0.96	–	3.70 ± 1.12
FGF21 (pg/ml)	152.41 ± 34.28	< 0.001	91.67 ± 11.66	–	114.31 ± 1.25

Data are presented as n (%) for categorical variables, median (IQR, interquartile range) or mean (SD, standard deviation) for continuous variables.

Differences across the three groups (NGT and two GDM subtypes) were compared using one-way ANOVA for normally distributed continuous variables, Kruskal–Wallis test for non-normally distributed continuous variables, or Chi-squared test for categorical variables. ^a^When P < 0.05, pairwise comparisons between the NGT group and each GDM group were made using the Tukey’s test, Dunn’s test, or Chi-squared test, respectively. P values for pairwise comparisons were adjusted using the Bonferroni correction.

At the second trimester, compared with the women in the NGT group, women of the two GDM subtypes showed higher circulating levels of blood glucose during the OGTT test and larger glycemic area under the curve (AUC) (all *P* < 0.05). The insulin levels at all the time points and AUC (for insulin) exhibited a statistically significant increase (*P* < 0.001) in the GDM-resistance group when compared with the NGT group. By contrast, insulin level at the 2^nd^ hour and AUC (insulin) were decreased in the GDM-dysfunction group (*P* < 0.001). The levels of insulin secretion indicators (HOMA2-β, Stumvoll I index) were higher in the GDM-resistance group but lower in the GDM-dysfunction group when compared with the NGT group (all *P* < 0.05). However, the insulin sensitivity indicators [HOMA2-S (*P* < 0.05) and ISI composite index (*P* < 0.05)] were decreased in the GDM-resistance group when compared with the NGT group. No statistically significant differences were found in the insulin sensitivity indicators between the women in the GDM-dysfunction group and the NGT group.

Before delivery, compared with women in the NGT group, women in the GDM-resistance group had larger AUC (for glucose) (*P* = 0.035). However, the blood glucose levels showed no statistically significant differences at all of the time points during the OGTT test between the GDM-resistance group and the NGT group (all *P >* 0.05). By contrast, women in the GDM-dysfunction group showed higher blood glucose levels during the OGTT test and larger AUC (for glucose) when compared with the NGT group (all *P* < 0.05). The overall tendency of other OGTT related indicators (insulin levels during OGTT test, AUC for insulin, insulin secretion and sensitivity indicators) in women of all the groups were similar from the 2^nd^ trimester to delivery. Women in the GDM-resistance group exhibited lower level of plasma LDL-C (*P* = 0.033) as compared with the NGT group. Whereas, we found no statistically significant differences of plasma HDL-C, CHO and TG levels between women in the GDM-resistance group and the NGT group. Women in the GDM-dysfunction group had comparable lipid profile with the NGT group.

The circulating levels of plasma FGF21 in women of the GDM-dysfunction group were similar with the NGT group. However, women in the GDM-resistance group had elevated plasma FGF21 levels when compared with the NGT group (*P* < 0.001).

### The Comparison of ISI Composite Index and Stumvoll I Index Between the Second Trimester and Before Delivery

**Figure 1:**
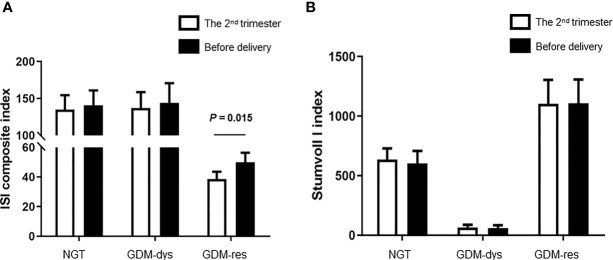
Comparison of ISI Composite and Stumvoll I Indexes between the 2^nd^ trimester and before delivery. **(A)** ISI Composite Index. **(B)** Stumvoll I Index. Data were analyzed by Mann-Whitney U test. NGT, *n* = 13; GDM-res, *n* = 17; GDM-dys, *n* = 9.

To investigate the alteration of insulin sensitivity and insulin secretion during pregnancy, we compared the ISI Composite Index and Stumvoll I Index between the second trimester and before delivery. The ISI composite index before delivery was improved when compared to that at the 2^nd^ trimester in the GDM-resistance group (*P* = 0.015, **Figure 1A**). The ISI composite index at these two time points had no statistically significant differences in the GDM-dysfunction group and the NGT group. Meanwhile, we did not observe any statistically significant differences of the Stumoll I index between the 2^nd^ trimester and before delivery in each group (**Figure 1B**).

### Linear Correlation Analysis Between the Clinical Indicators and Circulating FGF21 at Delivery

In order to find whether plasma FGF21 level is related with the clinical indicators, we performed linear correlation analyses. The plasma FGF21 levels were positively correlated with pre-BMI (r = 0.361, *P* = 0.028, **Table 2**) and negatively related to FBG (r = -0.718, *P <* 0.001, **Table 2**) and LDL-C (r = -0.438, *P* = 0.007, **Table 2**) at delivery. However, the plasma FGF21 levels were found negatively correlated with FBG (r = -0.574, *P* < 0.001, **Table 3**) and associated with the GDM-resistance group (r = 0.574, *P* < 0.001, **Table 3**) after adjusted by each other in the multiple linear regression. Plasma FGF21 concentrations had no linear relationships with GWG, CHO, TG, HDL-C.

**Table 2:** Linear correlation analysis between the clinical indicators and circulating FGF21 at delivery.

Variable	r	*P* ^a^
pre-BMI	**0.361**	**0.028**
GWG	-0.158	0.350
FBG	**-0.718**	**< 0.001**
CHO	-0.238	0.161
TG	-0.003	0.986
LDL-C	**-0.438**	**0.007**
HDL-C	-0.144	0.402

a Relationships between FGF21 and the clinical indicators were conducted using Pearson’s correlation analysis. n = 39. Bold values mean P < 0.05.

**Table 3:** Multiple linear regression analysis between the clinical indicators and circulating FGF21 at delivery.

Variable	r	B (95%CI)	*P* ^a^
pre-BMI	-0.214	-2.572 (-5.855~0.710)	0.120
LDL-C	-0.092	-3.054 (-10.375~4.268)	0.402
FBG	**-0.574**	**-60.207 (-80.478~-39.937)**	**< 0.001**
GDM-resistance	**0.574**	**43.864 (21.063~66.666)**	**< 0.001**

a Relationships between FGF21 and indicators correlated with FGF21 in linear correlation were conducted using multiple linear regression analysis. The NGT group is the reference in the classification variable. n = 39. Bold values mean P < 0.05.

### Relative Gene and Protein Expression of FGF21 Receptors and Signaling Pathways in vWAT

**Figure 2:**
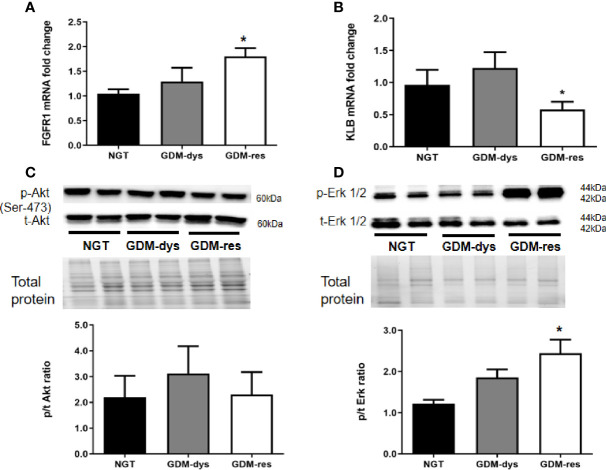
Relative gene and protein expression of FGF21 receptors and signaling pathways in vWAT. **(A, B)** Gene expression of FGFR1 and KLB. **(C, D)** Protein expression of phosphorylated/total Akt (Ser 473) and Erk1/2 (T202/204). *P < 0.05 *vs.* NGT group. Data were analyzed by a one-way ANOVA with Tukey *post hoc* test. NGT, *n* = 13; GDM-res, *n* = 17; GDM-dys, *n* = 9.

In order to further study the FGF21 signaling in target organs, we detected the relative gene and protein expression of FGF21 receptors and downstream signaling pathways in vWAT. Compared with the NGT group, gene expression of fibroblast growth factor receptor 1c (FGFR1c) was increased (*P* < 0.05, **Figure 2A**), while gene expression of β-Klotho (KLB) was decreased (*P* < 0.05) in the GDM-resistance group (**Figure 2B**).

Furthermore, we found no statistically significant differences in the phosphorylation level of Akt (Ser 473) among the three groups (**Figure 2C**). However, the phosphorylation level of Erk1/2 was increased in the GDM-resistance group (*P* < 0.05, **Figure 2D**) when compared with the NGT group. The Akt and Erk1/2 signaling was not significantly altered in the vWAT of GDM-dysfunction group as compared with the NGT group.

### Relative Expression of Genes Involved in Glucose Uptake, Insulin Sensitivity and Lipolysis in vWAT

**Figure 3:**
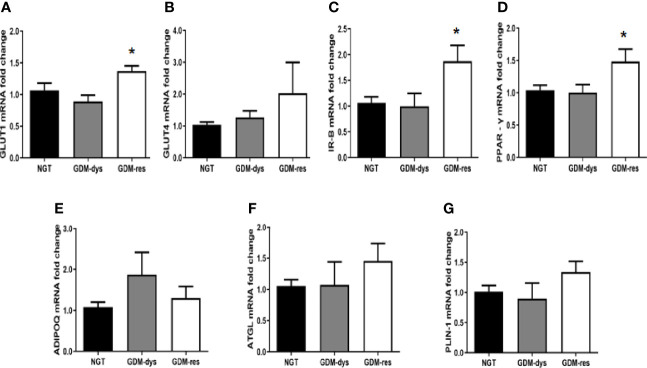
**|** Relative expression of genes involved in glucose uptake, insulin sensitivity and lipolysis in vWAT. **(A‒G)** Gene expression of GLUT1, GLUT4, IR-B, PPAR-γ, ADIPOQ, ATGL, PLIN-1. *P < 0.05 *vs.* NGT group. Data were analyzed by a one-way ANOVA with Tukey *post hoc* test. NGT, *n* = 13; GDM-res, *n* = 17; GDM-dys, *n* = 9.

We found higher mRNA expression of glucose transporter-1 (GLUT1), insulin receptor-β (IR-B) and peroxisome proliferators activated receptor-γ (PPAR-γ) (all *P* < 0.05, **Figures 3A, C, D**) in the vWAT of the GDM-resistance group when compared with that in the NGT group. The mRNA expression of glucose transporter-4 (GLUT4), adiponectin, C1Q and collagen domain containing (ADIPOQ), adipose triglyceride lipase (ATGL) and phosphoprotein perilipin (PLIN-1) of the GDM-resistance group showed no statistically significant differences as compared with the NGT group (**Figures 3B, E–G**). Meanwhile, in the GDM-dysfunction group, we observed no statistically significant differences in the mRNA expression of GLUT1, GLUT4, ADIPOQ, ATGL, PLIN-1, IR-B, and PPAR-γ when compared with the NGT group. In addition, we hardly detected the mRNA expression of FGF21 in vWAT (data not shown).

### Linear Correlation Analysis Between Circulating FGF21 at Delivery and Relative Expression of Genes Involved in Glucose Uptake, Insulin Sensitivity and Lipolysis

In **Table 4**, circulating FGF21 levels were positively correlated with the relative gene expression of GLUT1 (r = 0.383, *P* = 0.021), IR-B (r = 0.355, *P* = 0.042) and PPAR-γ (r = 0.402, *P* = 0.015). Plasma FGF21 concentrations at delivery had no linear relationships with the relative gene expression of GLUT4, ADIPOQ, ATGL and PLIN-1.

**Table 4:** Linear correlation analysis between circulating FGF21 at delivery and relative expression of genes involved in glucose uptake, insulin sensitivity and lipolysis in vWAT.

Variable	r	*P* ^a^
GLUT1	**0.383**	**0.021**
GLUT4	0.253	0.137
IR-B	**0.355**	**0.042**
PPAR-γ	**0.402**	**0.015**
ADIPOQ	0.058	0.749
ATGL	0.253	0.136
PLIN-1	0.117	0.443

a Relationships between FGF21 and relative expression of genes involved in glucose uptake, insulin sensitivity and lipolysis using Pearson’s correlation analysis. n = 39. Bold values mean P < 0.05.

## Discussion

We found that women of the GDM-resistance group showed improved insulin sensitivity before delivery compared with that at the 2^nd^ trimester. At the same time, increased plasma FGF21 concentrations and activated FGF21 signaling in the vWAT were found in the GDM-resistance group at delivery. Interestingly, these manifestations were not found in the women of the GDM-dysfunction group.

According to the classification of GDM subtypes in this study at the 2^nd^ trimester, decreased insulin sensitivity and increased insulin secretion were clinical characters of women in the GDM-resistance group, while insufficient insulin secretion and normal insulin sensitivity were the main manifestations of women in the GDM-dysfunction group. The GDM-mixed subtype has both characters above, and is manifested as a combination of these two physiologic and pathologic processes. A potential interaction effect may exist on FGF21 expression, so we excluded the GDM-mixed subtype. Compared with women in the NGT group, women in the GDM-resistance group showed higher pre-BMI, while women in the GDM-dysfunction group had comparable pre-BMI. Pregnant women with higher pre-BMI may be more prone to other metabolic syndrome during pregnancy.

Insulin resistance and obesity (21) affect the secretion of FGF21. Consistently, women in the GDM-resistance group but not the GDM-dysfunction group manifested increased plasma FGF21 concentrations compared with the NGT group in this study. A clinical study found human BMI were positively correlated with their circulating FGF21 concentrations (22), and the increased FGF21 concentrations compensate for the insulin resistance induced by obesity and other factors (7). These findings also explain the comparable circulating FGF21 levels between women in the GDM-dysfunction group and the NGT group, since they had similar pre-BMI and insulin sensitivity indexes.

Insulin resistance often accompanies with impaired FGF21 signal transduction (also referred as FGF21 resistance) in obese T2DM patients (23). However, the GDM-resistance group showed improved insulin sensitivity and elevated circulating FGF21 concentrations as compared with the NGT group at delivery. As we known, the increased circulating FGF21 levels were positively correlated with metabolic syndrome in obese population (24), because the physiological increased dose of circulating FGF21 helps to maintain insulin sensitivity in specific tissues during the early stages of these diseases (25). In animal studies, increased FGF21 expression in liver and adipose tissue was found in db/db mice (24). Besides, regular exercise helps to maintain metabolic homeostasis of the GDM-resistance women. Exercise increases the sensitivity of FGF21 in adipose tissue, then improves insulin sensitivity by sending humoral signals to coordinate multi-organs (26). Moreover, unlike T2DM patients, the participants of the GDM-resistance group in our study did not have severe metabolic disorders. At the beginning of the diagnosis of GDM, obstetricians often provide exercise instruction to control their weight gain. These may explain the inconsistency of the insulin resistance and FGF21 signal transduction between GDM-resistance women and T2DM patients.

Plasma FBG levels and the GDM-resistance subtype were independently correlated with plasma FGF21 concentrations. Rikke and colleagues (27) demonstrated that the physiological range of insulin increased serum FGF21 level through dose-dependent way during the euglycaemic hyperinsulinaemic clamp test. Studies found that FGF21 was negatively correlated with FBG after adjusted by age, sex, BMI and other confounding indexes (28), and was positively correlated with adiposity and fasting plasma insulin levels in healthy subjects after adjusted by BMI and age (22). These findings suggest that the elevated plasma FGF21 concentrations may associate with plasma insulin level, and thus play a role in improving plasma FBG.

We observed no FGF21 resistance in vWAT of the GDM-resistance group at delivery. FGFR1c and KLB are known to be the receptors of FGF21 (29). Interestingly, we found markedly increased FGFR1c expression, but reduced KLB expression in vWAT of the GDM-resistance group. Rikke and colleagues also found overweight/obesity led to decreased KLB but increased FGFR1c expression in WAT (27). FGF21 signaling through KLB in WAT may be primarily related with obesity, as decreased KLB expression was observed in WAT of obese mice (30), non-human primates fed with high-fat diet (31), and obese subjects with different levels of abnormal glucose homeostasis (32). However, FGFR1c is the predominant FGFR involved in FGF21 signaling (33). The increased FGFR1c expression could activate FGF21 signaling in a KLB-independent manner, and could compensate for the reduced KLB expression (34). Meanwhile, the GDM-resistance group showed increased Erk1/2 phosphorylation, and increased GLUT1, IR-B and PPAR-γ mRNA expression. The activation of Erk1/2 signaling in WAT could increase GLUT1 mRNA expression (7, 35), stimulate PPAR-γ transcriptional activity, promote insulin-independent glucose uptake, improve insulin sensitivity and inhibit lipolysis (36, 37). Meanwhile, we found FGF21 concentration was positively correlated with the relative expression of GLUT1, IR-B and PPAR-γ by the linear regression analysis. These data suggest that the FGF21 signaling pathway was activated in the vWAT of the GDM-resistance group.

We could not detect the mRNA expression of FGF21 in the vWAT, and failed to found significant differences in the mRNA expression of lipolysis genes and ADIPOQ in the vWAT of the GDM-resistance group. Sara and colleagues (38) also found that basal FGF21 mRNA expression was hardly detected in adipose tissue of young men, while the expression of FGF21 was significantly increased under supraphysiological insulin level during hyperinsulinemic euglycemic clamp. FGF21 inhibits lipolysis by reducing expression of lipid droplet-associated phosphoprotein, but not affects expression of lipolysis regulatory genes (39). FGF21 increases plasma adiponectin levels by enhancing both its gene transcription and protein secretion in adipocytes (40), however, many other factors could negatively mediate the expression of ADIPOQ, such as pro-inflammatory cytokines, endoplasmic reticulum stress, and oxidative stress (41).

For the potential medical applications of FGF21 treatment, systemic administration of FGF21 has an effect on improving insulin sensitivity (42). The injection of recombinant FGF21 improved blood glucose tolerance and insulin sensitivity in leptin deficient OB/OB mice and DIO mice (insulin-resistant mice) (43). With the in-depth understanding of the pathophysiology of FGF21 in the GDM subtypes, the FGF21-targeted prevention and treatment approaches may become a new option for specific GDM subtype.

The limitations of our research included that it is a single-center study with limited sample size, since the included participants must have complete data of OGTT and simultaneous insulin-release test at the 2^nd^ and 3^rd^ trimesters, and the GDM mixed subtype was excluded. We will expand the sample size and establish a cohort study of pregnant women in the future. Furthermore, we failed to collect plasma and vWAT samples of pregnant women at the 2^nd^ trimester, so that we could not delineate the dynamic changes of circulating FGF21 concentrations and FGF21 signaling in the vWAT during pregnancy. Additionally, we lack the data of hyperinsulinemic and hyperglycemic clamp test during pregnancy, which prevents us from clarifying the interaction between circulating FGF21 concentrations and blood glucose/insulin levels.

**Figure 4:**
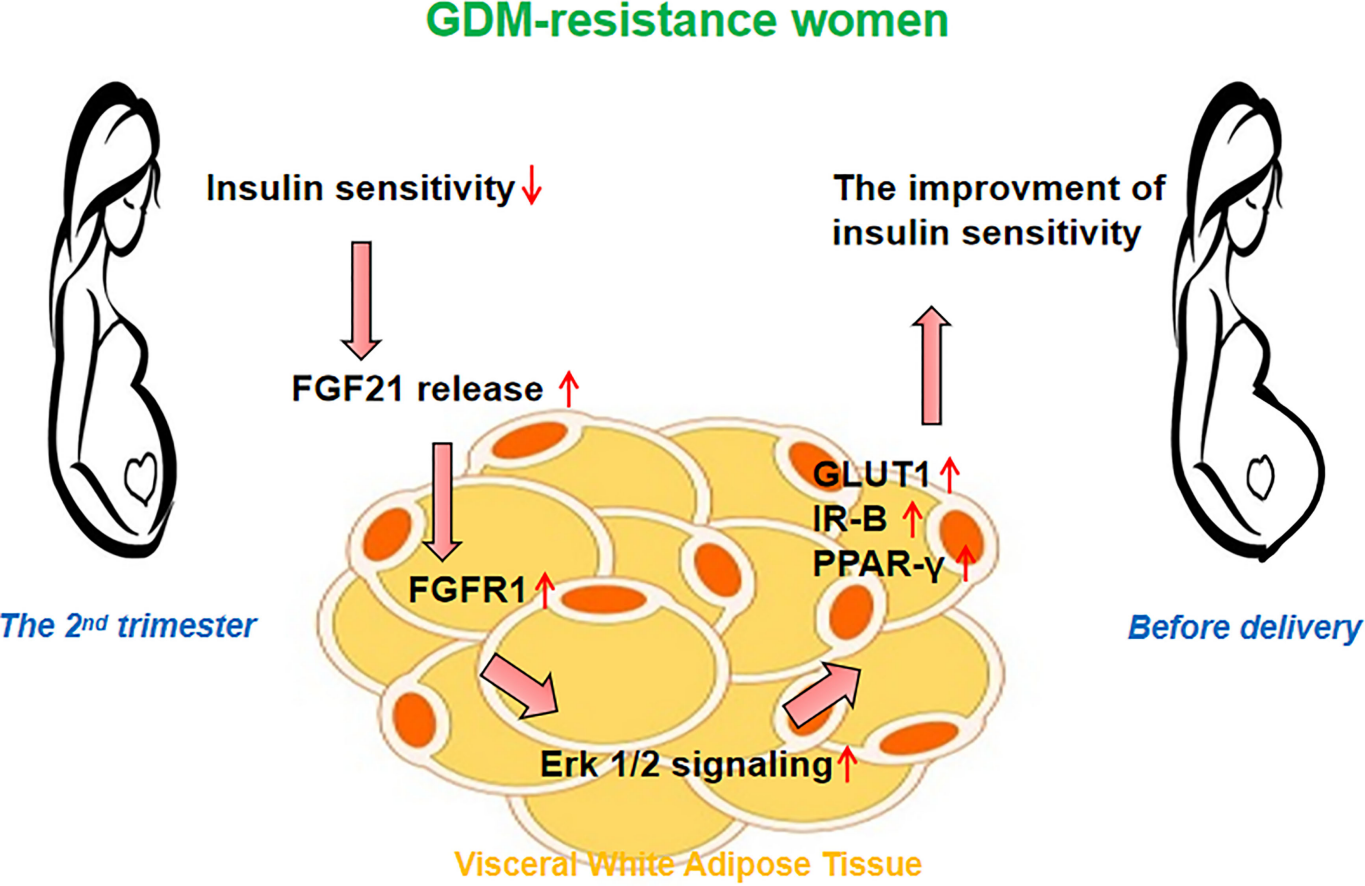
Hypothesis on the role of FGF21 signaling in the visceral white adipose tissue (vWAT). The improved insulin sensitivity in women of the GDM-resistance group may be associated with the increased plasma FGF21 level and activated FGF21 signaling in vWAT.

In conclusion, we found women with GDM heterogeneity exhibited different insulin sensitivity alteration patterns from the 2nd trimester to delivery. The improved insulin sensitivity in the women of the GDM-resistance group may be associated with the increased FGF21 level and activated FGF21 signaling in the vWAT (**Figure 4**). Our results point out a new direction for understanding the function of FGF21 in GDM women. Based on the etiology and pathogenesis of GDM heterogeneity, the dynamic balance of plasma FGF21 may help to understand the insulin sensitivity alteration patterns during pregnancy.

## Data Availability Statement

The datasets presented in this study can be found in online repositories. The names of the repository/repositories and accession number(s) can be found in the article/**Supplementary Material**.

## Ethics Statement

The studies involving human participants were reviewed and approved by The Ethics Committee of the First Affiliated Hospital of Xi’an Jiaotong University (XJTU1AF2019LSL-007). The patients/participants provided their written informed consent to participate in this study.

## Author Contributions

NW, LS, and WC designed the work presented by the article. NW, LS, and BS completed the experiment and drafted and revised the article. HG, YJ, QR, MW, YM, and HC collected the data and revised the article for critically important content. LS and WC final approved of the version to be published. All authors contributed to the article and approved the submitted version.

## Funding

We acknowledge grant funding of the Natural Science Foundation of Shaanxi Province (No. 2020GXLH-Y-029, 2019JQ069, 2019JM262), the Bethune-Merck Diabetes Research Foundation (No. G-X-2019-056), the Clinical Research Award of the First Affiliated Hospital of Xi’an Jiaotong University, China (No. XJTU1AF-CRF-2019-007), the Natural Science Foundation of China (No. 81801459; No. 81741079; No. 82071732), the Natural Science Foundation for Postdoctoral Scientists of China (No. 2018M641001, No. 2016M600799).

## Conflict of Interest

The authors declare that the research was conducted in the absence of any commercial or financial relationships that could be construed as a potential conflict of interest.

## Publisher’s Note

All claims expressed in this article are solely those of the authors and do not necessarily represent those of their affiliated organizations, or those of the publisher, the editors and the reviewers. Any product that may be evaluated in this article, or claim that may be made by its manufacturer, is not guaranteed or endorsed by the publisher.
