# Supplementary material for: Association of Elevated Plasma FGF21 and Activated FGF21 Signaling in Visceral White Adipose Tissue and Improved Insulin Sensitivity in Gestational Diabetes Mellitus Subtype: A Case-Control Study
Source: Front Endocrinol (Lausanne). 2021 Nov 29;12:795520. doi: 10.3389/fendo.2021.795520 (PMC8667891; doi:10.3389/fendo.2021.795520)
Supplement: Supplementary file 1 [file Table_1.docx]

**Supplementary Table 1.** The sequences of primers for the tested genes

| NCBI Ref. Sequence | Primer | Subsequence |
| --- | --- | --- |
| [NM_001177800.2](https://www.ncbi.nlm.nih.gov/nuccore/NM_001177800.2) | ADIPOQ-F | CTGGTGAGAAGGGTGAGAAAG |
|  | ADIPOQ-R | ACTCCGGTTTCACCGATGTC |
| [NM_001274304.1](https://www.ncbi.nlm.nih.gov/nuccore/NM_001274304.1) | GLUT1-F | GCGGAATTCAATGCTGATGAT |
|  | GLUT1-R | CAGTTTCGAGAAGCCCATGAG |
| [NM_001042.3](https://www.ncbi.nlm.nih.gov/nuccore/NM_001042.3) | GLUT4-F | CGTGGGCGGCATGATT |
|  | GLUT4-R | CCAGCATGGCCCTTTTCC |
| [NM_019113.4](https://www.ncbi.nlm.nih.gov/nuccore/NM_019113.4) | FGF21-F | AGTGGAGCGATCCATACAGG |
|  | FGF21-R | ACTCCAGTCCTCTCCTGCAA |
| [NM_001174063.2](https://www.ncbi.nlm.nih.gov/nuccore/NM_001174063.2) | FGFR1c-F | CCCGTAGCTCCATATTGGACA |
|  | FGFR1c-R | TTTGCCATTTTTCAACCAGCG |
| [NM_175737.4](https://www.ncbi.nlm.nih.gov/nuccore/NM_175737.4) | KLB-F | CCATCCGCCGAGGATTATTT |
|  | KLB-R | GGTAGTGGGTGACTTTCATTCT |
| [NM_001145311.2](https://www.ncbi.nlm.nih.gov/nuccore/NM_001145311.2) | PLIN1-F | CCCCCTGAAAAGATTGCTTCT |
|  | PLIN1-R | GGAACGCTGATGCTGTTTCTG |
| [NM_020376.4](https://www.ncbi.nlm.nih.gov/nuccore/NM_020376.4) | ATGL-F | CGAGAATGTCATTATATCCCACTTCA |
|  | ATGL-R | TGAGCCCACAGTACACGGG |
| [NM_000208.4](https://www.ncbi.nlm.nih.gov/nuccore/NM_000208.4) | IR-B-F | AGGAGTCCTCGTTTAGGAAG |
|  | IR-B-R | AGGAAGTGTTGGGGAAAG |
| [NM_001330615.4](https://www.ncbi.nlm.nih.gov/nuccore/NM_001330615.4) | PPAR-γ-F | TCAGGGCTGCCAGTTTCG |
|  | PPAR-γ-R | CCCTCGGATATGAGAACCC |
| [NM_000942.5](https://www.ncbi.nlm.nih.gov/nuccore/NM_000942.5) | Cyclophilin-F | TCTGCACTGCCAAGACTGAG |
|  | Cyclophilin-R | TCGAGTTGTCCACAGTCAGC |
